# High-speed visualization of ultrasonic emulsification in microgravity: interactions between cavitation dynamics and liquid–liquid interface topology

**DOI:** 10.1016/j.ultsonch.2026.107895

**Published:** 2026-05-20

**Authors:** Jakob Mali, Žan Boček, Jernej Ortar, Sara Poropatič, Leon Čepin, Nejc Bertoncelj, Matevž Dular

**Affiliations:** a University of Ljubljana, Faculty of Mechanical Engineering, Aškerčeva cesta 6, 1000 Ljubljana, Slovenia; b University of Ljubljana, Faculty of Natural Sciences and Engineering, Aškerčeva cesta 12, 1000 Ljubljana, Slovenia; c University of Ljubljana, Faculty of Electrical Engineering, Tržaška cesta 25, 1000 Ljubljana, Slovenia

**Keywords:** Zero Gravity, Emulsion, Cavitation

## Abstract

Ultrasonic emulsification is a widely used technique for generating fine dispersions of immiscible liquids, yet its underlying mechanisms remain only partially understood, particularly under reduced-gravity conditions. In the absence of gravity, buoyancy-driven phase separation vanishes, fundamentally altering the organization of liquid–liquid interfaces and their interaction with cavitation structures.

This study investigates the dynamics of ultrasonic emulsification in microgravity using high-speed visualization during parabolic flight experiments, with systematic variation of oil–water volume ratios.

The results reveal that microgravity leads to capillarity-dominated interface configurations, where the position and stability of the oil–water interface become less predictable compared to normal gravity conditions. As a consequence, sustained interaction between the cavitation zone and the interface is more difficult to achieve. Since such interaction was previously identified as the key mechanism driving droplet breakup, its reduction leads to slower emulsification rates and lower final emulsion homogeneity.

Image analysis shows that emulsification under normal gravity proceeds faster and produces more homogeneous dispersions, while microgravity conditions lead to delayed evolution and lower overall homogeneity.

The findings demonstrate that ultrasonic emulsification in microgravity is governed by an interplay between interface topology and cavitation dynamics and highlight the importance of controlled interface positioning for enabling efficient multiphase fluid processing in space environments.

## Graphical abstract

**Figure:**
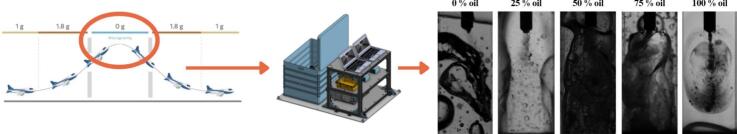

## 1 Introduction

Human exploration beyond Earth orbit is entering a phase focused on the development of sustainable and long-term operational capabilities. Establishing a continuous human presence on the Moon and preparing for future missions to Mars require substantial advances across a broad range of scientific and engineering disciplines. To address these challenges systematically, the European Space Agency (ESA) has, as a part of its Roadmap, identified 26 research topics that are essential for enabling sustainable space operations [1]. Among these, Physics of Emulsions (ID no. 15) has been recognized as an important area requiring targeted investigation.

Emulsions are heterogeneous mixtures consisting of two immiscible liquids, where one phase is dispersed within the other in the form of droplets. Such systems are widely used across numerous industrial sectors, including food and beverage processing, pharmaceuticals, cosmetics, agriculture, and medicine, where their physical properties and stability are critical for product performance [2], [3], [4]. The formation of emulsions requires the application of significant energy to overcome interfacial tension and fragment one liquid into small droplets within the continuous phase [2]. Various techniques have been developed to supply this energy, ranging from mechanical approaches such as high-pressure homogenization [5], rotor–stator mixing [6] to acoustic [3] and hydrodynamic cavitation [7].

Among these techniques, ultrasonic emulsification has gained considerable attention due to its ability to generate fine and relatively uniform emulsions while often requiring less energy and lower surfactant concentrations compared with conventional mechanical methods [2], [3]. In ultrasonic systems, acoustic waves generated by oscillating piezoelectric transducers propagate through the liquid, producing cavitation − the growth, oscillation, and violent collapse of vapor or gas bubbles within the fluid. The collapse of these bubbles generates intense localized phenomena such as microjets, shock waves, and strong microstreaming flows, which enhance mixing and promote droplet breakup [3].

Despite extensive application of ultrasonic emulsification, the detailed physical mechanisms governing droplet formation remain only partially understood. Current understanding describes the process as a sequence of cavitation-driven interactions between bubbles, liquid phases, and the oil–water interface [8], [9]. Previous investigations have shown that during bulk emulsification complex intermediate structures may form, such as water-in-oil or multiple emulsions within the oil phase, which subsequently disperse into the surrounding liquid and break down into a final oil-in-water emulsion [10]. These transformations are primarily driven by hydrodynamic effects associated with cavitation bubble collapse, including liquid jets and shock waves that interact with the oil–water interface [8], [9], [11].

The dynamics of these interactions depend strongly on the spatial relationship between cavitation bubbles and the liquid–liquid interface. Experimental studies have demonstrated that the direction of jetting produced by collapsing bubbles is governed by the density contrast between phases: when bubbles grow in the denser phase (typically water), the resulting jet tends to move away from the interface, whereas bubbles forming in the lighter phase (oil) generate jets directed toward the interface [11]. Building on this understanding, more recent work has focused on detailed visualization of cavitation events near liquid–liquid interfaces, revealing how single bubbles can deform droplets, trigger interfacial instabilities, or initiate droplet fragmentation depending on parameters such as bubble size, oil viscosity, and the relative distance between bubble and interface [11].

While these studies have significantly advanced the understanding of cavitation-induced emulsification at the scale of individual bubbles, comparatively less attention has been devoted to bulk ultrasonic emulsification systems operating with practical ultrasonic horns. In such systems, cavitation develops in dense bubble clusters and interacts with the oil–water interface in a highly dynamic and spatially complex manner, making the governing mechanisms difficult to observe and characterize experimentally.

The transition from single-bubble mechanics to the bulk emulsification process observed in practice occurs progressively. Individual cavitation events near the oil–water interface initially follow the jetting directions governed by the local density contrast, as described above. However, under continuous ultrasonic irradiation these individual collapse events do not occur in isolation — they rapidly combine into a collective cavitation cluster that forms and grows at the horn tip [8], [9]. The energy released by this cluster drives acoustic streaming, generating a recirculating bulk flow within the vessel that extends well beyond the immediate vicinity of individual bubble collapse events[12]. This bulk flow plays a critical role in the emulsification process: it actively transports oil droplets and oil-phase fragments toward the cavitation zone near the horn tip, where the intense pressure gradients and shockwaves associated with bubble collapse break them down further into finer emulsion droplets. This transport mechanism accelerates the overall emulsification rate and improves the dispersion of the oil phase, as droplets recirculated into the cavitation zone are subjected to repeated breakup events rather than a single interaction. The macro-scale emulsification behavior observed in the present study is therefore best understood not as a departure from single-bubble physics, but as its collective and self-reinforcing manifestation at the bulk scale, where flow-mediated transport and cavitation-driven breakup act together as coupled and mutually enhancing processes.

**Fig. 1:**
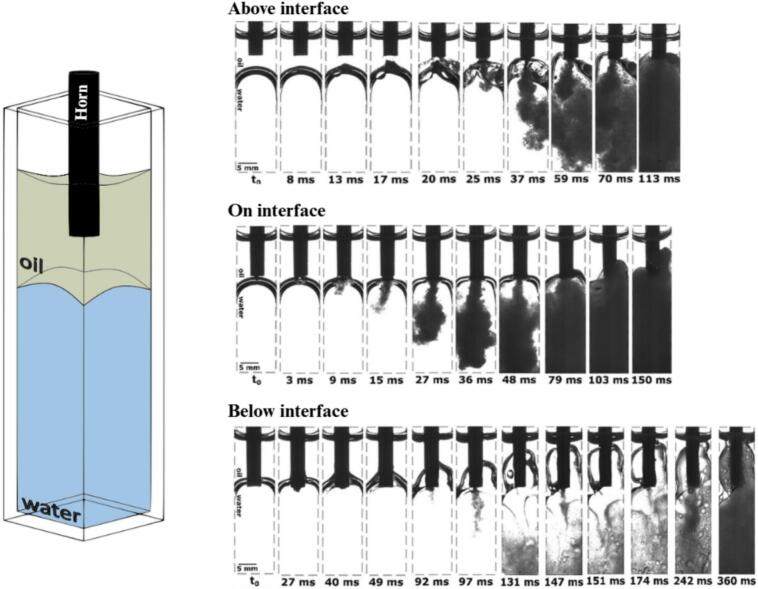
Ultrasonic emulsification with the ultrasonic horn positioned at various distances from the oil–water interface (adapted from our previous study [13]).

In previous laboratory investigations, it has been demonstrated that the position of the oil–water interface relative to the ultrasonic horn tip represents a key parameter controlling both the efficiency of ultrasonic emulsification and the properties of the resulting emulsion (Fig. 1) [13].

The results (Fig. 1) show that the most effective and stable emulsification occurs when the horn tip is located directly within the oil–water interface, or when a thin oil layer is present immediately beneath the horn followed by the interface and bulk water. Under these conditions, cavitation continuously disrupts the interface, producing a strong and wide emulsion stream and a higher initial rate of oil incorporation into the water phase. In contrast, a separation of only a few millimetres between the horn tip and the interface results in poor formation of a fine emulsion: in such cases, emulsification is delayed, intermittent, or weaker because the cavitation zone does not simultaneously interact with both liquid phases. Similarly, when the horn is placed too far below the interface, emulsification becomes less efficient and produces a coarser emulsion, as large oil fragments are torn from the interface in regions with insufficient cavitation to break them down. These observations confirm that efficient ultrasonic emulsification requires precise control of horn placement to ensure sustained contact between cavitation, the interface, and both phases, making interface position a decisive parameter in process optimization.

In microgravity conditions, the absence of buoyancy-driven phase separation leads to poorly defined and dynamically evolving oil–water interfaces, making the fundamental understanding and control of emulsification processes directly aligned with one of ESA’s priority research fields – Soft or complex matter research, specifically physics of emulsions [1].

Research on emulsions in microgravity has predominantly focused on stability mechanisms by eliminating gravity-driven effects such as creaming and sedimentation. It has been shown that, in the absence of buoyancy, droplet interactions and coalescence pathways are significantly altered, with emulsion ageing governed by internal dynamics rather than phase separation [14]. Furthermore, it has been demonstrated that microgravity conditions enable direct observation of droplet-scale dynamics, which are otherwise obscured under normal gravity due to sedimentation and convection effects [15].

To facilitate such investigations, specialized experimental platforms have been developed, allowing controlled studies of emulsions under reduced gravity conditions, including parabolic flight setups [16]. In addition, experiments conducted under simulated microgravity have confirmed that emulsion stability is primarily dictated by interfacial properties and kinetic processes when gravitational effects are suppressed [17]. These studies consistently report differences in droplet size distribution, coalescence rates, and long-term structural evolution compared to terrestrial conditions.

Overall, microgravity has been established as a powerful environment for studying the fundamental physics of emulsion stability, free from gravitational artifacts. Most of existing microgravity research focuses on processes, such as coalescence and ageing, with significantly less attention given to the mechanisms governing initial steps in emulsion formation – for the case of microgravity conditions these remain largely unexplored.

The present paper investigates how oil–water interface reorganizes itself in microgravity conditions and how this subsequently influences the emulsification process. To achieve this, the experimental setup was flown aboard a specially modified aircraft performing a zero-gravity parabolic flight maneuver. The results demonstrate a clear dependence of both the emulsification process and the final emulsion quality on the initial conditions of the experiment, determined by the presence or absence of gravity and the oil-to-water volume ratio within the cuvette. These findings are essential for advancing the understanding of emulsification processes and for informing the design of emulsion preparation and production systems for future space missions.

### 1.1 Oil–water interface in microgravity

The equilibrium and dynamics of immiscible fluid interfaces are largely governed by gravity through buoyancy and hydrostatic pressure gradients. In contrast, under microgravity (0 g) conditions, gravity-driven forces vanish and the system is instead controlled by interfacial tension, viscous stresses, inertia, and wetting interactions with confining boundaries.

Under normal gravity and experimental scales (L), the Bond number (Eqn. (1) [18]:

(1) $$\mathrm{Bo}=\frac{\Delta \rho gL^{2}}{\sigma },$$

is usually of an order of unity, implying a competition between gravity (g) and surface tension (σ) that stabilizes stratified configurations with the lighter phase above the heavier one (Δρ giving the density difference between the two phases). In microgravity, however, g ≈ 0 and as a consequence Bo ≈ 0. Hence the interface is no longer governed by the gravity, but by capillarity and boundary conditions – the system strives toward configurations that minimize total interfacial free energy. When fluid is in motion (velocity during reorientation of the interface (U)), the ratio between the viscous (μ) drag forces and the surface tension forces acting across an interface between the two immiscible liquids is given by the Capillary number (Eqn. (2) [18]:

(2) $$\mathrm{Ca}=\frac{\mu U}{\sigma }$$

The relative importance of inertial forces with respect to surface tension and viscous forces is characterized by the Weber (Eqn. (3) and Reynolds numbers (Eqn. (4), respectively [19]:

(3) $$\mathrm{We}=\frac{\rho U^{2}L}{\sigma }$$

(4) Re=ρULμ

For the low velocities and small length scales typical of the present experiment, both Re and We are usually small, indicating laminar flow and capillary-dominated interfacial dynamics. In this regime, interface curvature is set primarily by surface tension and confinement rather than inertia.

According to Eqn. (2), Ca≪1. Under these conditions, the interface relaxes toward a configuration that minimizes total interfacial free energy.

In confined geometries (such as in the present study), a key parameter that becomes particularly important for the interfacial topology in microgravity is the wetting condition at the solid boundary (cuvette walls). It can be characterized by the equilibrium contact angle and determines which phase preferentially coats the container walls. If one fluid wets the solid more strongly, it will tend to form thin films along the boundaries, while the non-wetting phase retracts into the bulk to minimize its contact with the solid.

## 2 Experimental set-up

### 2.1 Test section

**Fig. 2:**
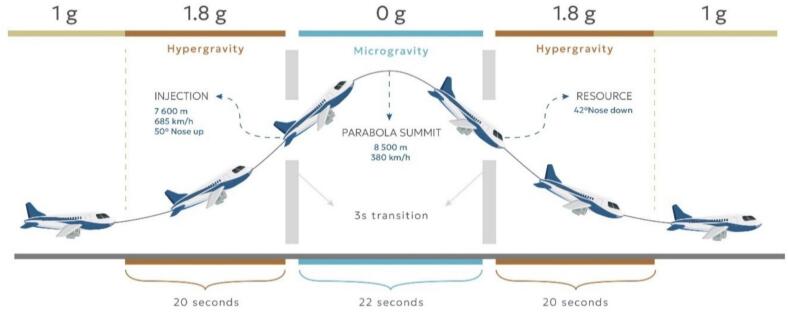
Zero gravity parabolic maneuver.

The experiment was flown on the Novespace Air Zero G airbus A310, which is a specially converted airframe that allows precise execution of the parabolic maneuver as shown in Fig. 2.

In general, the parabolic maneuver is composed of five periods [20]. Firstly, aircraft climbs to the cruising altitude of around 6000 m above sea level. At this time the airframe is exposed to the normal gravity – 1 g (9.81 m/$s^{2}$). At this time, the experimental crew can access the setup and prepare for the zero-gravity maneuver. That starts with the rapid ascend, that lasts around 20 s and at that time the experiment is exposed to hyper gravity conditions at around 1.8 g. Next, the most relevant phase follows – zero gravity phase. This is the main, experimental part of the parabolic flight, and it lasts approximately 22 s. During this phase, the interior of the aircraft is exposed to local weightlessness conditions. After the 0 g phase, aircraft once again enters the hyper-gravity phase during the pull-out maneuver. One parabola is complete once the aircraft enters the normal flight and is exposed to normal gravitational conditions. At the steady flight between the parabolas, that lasts around one minute and half, experimental setup can be approached by experimental crew; samples can be changed and stored, and the whole procedure is repeated in the next parabola. Each European Space Agency parabolic campaign has three flying days and during each day, 31 parabolic maneuvers are flown − one test parabola and 30 experimental parabolas. In total, that accumulates 93 parabolas and approximately 30 min of zero gravity exposure.

**Fig. 3:**
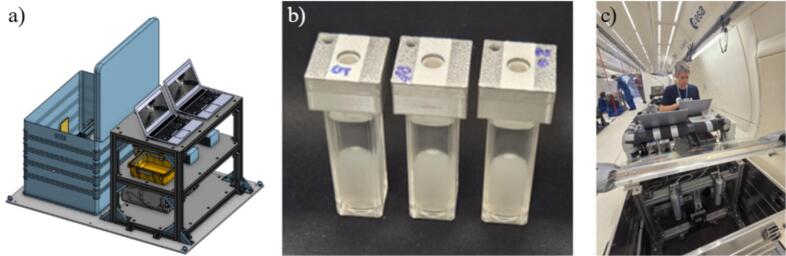
Experimental setup. (a) CAD model of the experimental station, (b) the cuvettes, (c) the setup during installation in the aircraft.

The characteristics of the experimental platform require that all research equipment be secured inside aluminum housing, which is attached to the floor of the aircraft via guides. The main parts of the setup are presented in Fig. 3.

As seen in CAD model (Fig. 3a) the housing was composed of two main parts, watertight Zarges box, where the cavitational emulsification took place during 0 g phase and the supporting rack, which hosted all of the experimental equipment. The design allowed two experiments to be run simultaneously.

#### 2.1.1 Cuvette

The Zarges box hosted polystyrene cuvettes (Fig. 3b). Standard cuvettes for photometric measurements with inner dimensions of 10 × 10 × 45 mm were used. They were filled with 3.6 mL of liquid at different oil–water ratios. Each cuvette was equipped with specially designed cap, that included silicon valve. This provided water tightness of the cuvette before and during insertion and also after removal of the tip of ultrasonic horn.

#### 2.1.2 Ultrasonic horn

For the generation of cavitation we used two Hielscher UP200St ultrasonic homogenizers equipped with titanium S26d2 horn tips with 2 mm diameter. The device has a maximum rated electrical power of 200 W. The oscillation frequency was fixed at 26 kHz and the amplitude was set to 50% of the device maximum, corresponding to a maximum delivered electrical power of approximately 100 W. The absolute tip displacement at this amplitude setting was measured independently from the experiment in microgravity. According to the manufacturer, the maximum peak-to-peak displacement at the transducer base is 70 µm, with the actual tip displacement being further amplified by the geometry of the S26d2 sonotrode to roughly 100 µm. No separate acoustic power measurement, such as calorimetry, was performed during the parabolic flight campaign, as the primary focus of the experimental setup was high-speed visual observation within the strict mass and volume constraints of the parabolic flight platform.

#### 2.1.3 Cameras

The analysis of the observed phenomena is based on visualization. We achieved that with two Photron high speed SA-Z cameras, that were upgraded with optional solid state drive, FAST drive. Both cameras were equipped with 105 mm lens. Frame rate was set at 50,400 fps, shutter speed at 18.23 µs and resolution at 384 × 880 px. Emulsification phenomena begins right after the horn is switched on, so the recording duration was set at 2.264 s.

#### 2.1.4 Illumination

With high-speed visualization one of the important parameters is the right illumination. In our set up we used two high-power LED panels that were placed inside the Zarges box. Input power per LED panel was 36 W. This provided sufficient brightness for the chosen shutter speed and was within thermal constraints.

#### 2.1.5 Triggering

All of the subsystems were connected together via triggering system. Triggering architecture was designed to be camera-centered. During every single parabola the experiment was triggered by mechanical trigger switch that started the recording. Simultaneously each camera provided positive output signal that triggered downstream systems − ultrasonic horn and illumination.

#### 2.1.6 Data acquisition

Primary data was stored onboard the cameras. Each FAST drive has a capacity of 4 TB. In addition to the visualization data, we recorded environmental data via the Raspberry Pi “environmental data acquisition” subsystem. Here we recorded ambient temperature inside the Zarges box, liquid temperature was measured continuously in an unused sample cuvette, ambient pressure inside the Zarges box, acceleration and angular velocity of the experimental rack. This secondary data was timestamped and saved to CSV file on a USB. The primary focus of environmental data acquisition subsystem was to serve as a backup, baseline data with which we could resolve any anomalies within the primary data.

### 2.2 Materials

We used distilled water. Its density was 998 kg/m^3^, its dynamic viscosity was 1 mPas and surface tension was 72.8 mN/m. In addition, we used AK 100 silicone oil (Wacker Chemie AG). Its density was 967 kg/m^3^, dynamic viscosity was 100 mPas and vapour pressure less than 10 Pa. The ambient temperature during the experiments was maintained at 20 °C, and the sonication time was sufficiently short that no significant temperature increase was observed.

### 2.3 Test conditions

In microgravity, the absence of buoyancy fundamentally alters the organization of immiscible fluid interfaces. As described in Section 1.1 the interface behavior and final position is governed by capillarity and wetting. The cuvette walls are made of polystyrene, a material that is generally hydrophobic and preferentially wetted by oils rather than water. The experiments were conducted in a fully filled cuvette, imposing strong geometric confinement of the interface, which relaxed to a quasi-static, constant-mean-curvature configuration in which oil wets the walls and water forms an interior domain.

It is important to estimate the time scale of the relaxation. Once the microgravity conditions are established, and in the absence of external forcing (for example shaking), the reorganization of the oil–water interface is driven by the balance between capillarity forces, which drive the system toward a minimum-energy configuration, and resistive effects originating from viscous dissipation or inertia. The characteristic relaxation times of these two mechanisms can be independently estimated to determine the timescales for the interfacial adjustment process. For the case of viscous dissipation:

(5) $$\frac{\sigma }{L}\propto \mu \frac{U}{L}\overset{\mathrm{yields}}{\to }U\;\frac{L}{t_{c}}\;\frac{\sigma }{\mu }\overset{\mathrm{yields}}{\to }t_{c,vis}\;\frac{\mu L}{\sigma }$$

and for the case of inertia:

(6) $$\frac{\sigma }{L}\propto \rho U^{2}\overset{\mathrm{yields}}{\to }U\;\frac{L}{t_{c}}\;\sqrt{\frac{\sigma }{\rho L}}\overset{\mathrm{yields}}{\to }t_{c,inn}\;\sqrt{\frac{\rho L^{3}}{\sigma }}$$

Two characteristic timescales are relevant in the present case: the time associated with the formation of a water droplet in the interior of the cuvette, and the time required for oil to wet the cuvette walls.

In the present experiment the largest length scale can be associated with the wetting along the entire cuvette height (L = 50 mm). This, with μ =100 mPas and σ ≈ 0.07 N/m, yields times of: t_c,vis_ ≈ 0.1 s and t_c,inn_ ≈ 1.5 s. The latter, longer characteristic time, suggests that from roughly 2 s after the onset of microgravity and in the absence of external driving forces the interface configuration should remain steady.

Realistically, full interfacial equilibration could take longer during experiments due to factors such as surface contamination and g-jitter. The g-jitter levels characteristic of the Novespace Airbus A310 Zero-G platform are ± 0.02 g in the longitudinal axis, ±0.01 g in the lateral axis, and ± 0.03 g in the vertical axis, as reported in the platform specifications provided by Novespace. During the initial reorganization of the oil–water interface following the onset of microgravity, these residual accelerations may contribute additional perturbations that slow the establishment of the equilibrium configuration. However, once a quasi-static interfacial equilibrium has been reached, residual accelerations at these levels are insufficient to fully disrupt and reorganize the interface. While g-jitter may introduce small local deformations, the capillary restoring forces governing the equilibrium configuration are sufficiently strong to maintain the overall interfacial topology. The 22-second microgravity window available during a parabolic flight campaign represents a fundamental constraint of the experimental platform, and the experimental protocol was designed to make the best possible use of it. Sonication was therefore initiated during the final 3 s of each microgravity period, allowing approximately 19 s for the interface to reorganize following the onset of weightlessness.

To assess the actual state of the interface prior to sonication, the first frames of each high-speed visualization recording were examined. Since the camera recording was initiated before the activation of the ultrasonic horn, these frames provide a direct observation of the interfacial configuration immediately prior to sonication. In all cases, the interface appeared smooth and stable, with no visible large-scale motion, indicating that a quasi-static equilibrium had been established prior to the onset of emulsification. We therefore consider the interfacial configuration at the moment of horn activation to be sufficiently quasi-static for the purposes of the present study. It should be noted that achieving a longer settling period would require an extended microgravity exposure beyond what parabolic flight can offer.

## 3 Results and discussion

Experiments at different water – oil volume ratios are presented and compared, from 100% water, then is steps of 25% to 100% oil. Variations in the water–oil volume ratio primarily affect the size of the bulk (interior) water region, while the oil remains boundary-attached across all ratios considered.

### 3.1 0% Oil, 100% water

The first example represents cavitation dynamics in a single-phase liquid, in our case distilled water without the presence of oil. This configuration serves as a reference case for understanding the hydrodynamic behavior generated by the ultrasonic horn in microgravity conditions before introducing a liquid–liquid interface.

**Fig. 4:**
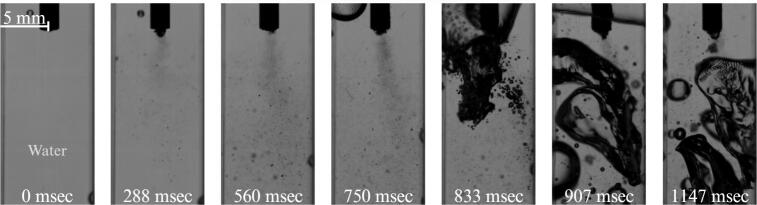
Cavitation in water at 0 g conditions (See also video in Supplementary 1).

Fig. 4 presents a sequence of seven consecutive frames illustrating the development of cavitation within the water-filled cuvette. Immediately after activation of the ultrasonic horn, a dense cluster of cavitation bubbles forms at the tip of the sonotrode. As the number of bubbles increases, the cluster reaches a critical density and evolves into a well-defined cavitation “stream” extending from the horn tip toward the bottom of the cuvette.

As microgravity conditions are established the gas in a partially filled reservoir slowly begins to migrate into the center of the reservoir. Other processes, such as g-jitter, oscillations of the setup, but primarily cavitation stream act as additional drivers of fluid motion within the cuvette. The continuous generation and collapse of bubbles produce strong acoustic streaming, which transports cavitation bubbles downward along the stream axis. The resulting momentum transfer induces a recirculating flow inside the cuvette, characterized by downward motion along the stream and compensating upward flow along the side walls. In the absence of gravity, this recirculating flow significantly influences the motion of gas inclusions present in the liquid. As shown in Fig. 4, an air bubble initially located above the horn can be transported downward by the induced flow field. Once it reaches the vicinity of the horn tip, the bubble interacts with the cavitation region and rapidly fragments into smaller bubbles that disperse into the surrounding liquid.

Such behavior contrasts with observations under normal gravitational conditions. On Earth, buoyancy forces dominate the motion of larger gas bubbles, causing them to rise away from the cavitation region and preventing sustained interaction with the ultrasonic horn. In microgravity, however, the absence of buoyancy allows the flow generated by cavitation to dominate bubble transport, enabling direct contact between the large air bubble and the horn tip and leading to its rapid breakup.

The reference (water only) case demonstrates that, in microgravity conditions, the flow field generated by ultrasonic cavitation can govern the transport and fragmentation of larger gas bubbles within the liquid. Understanding this baseline hydrodynamic behavior is essential for interpreting the more complex interactions that occur once a liquid–liquid interface is introduced in the subsequent experiments.

### 3.2 25% Oil, 75% water

When a small amount of oil is introduced into the system, the interfacial topology within the cuvette changes significantly compared with the single-phase reference case. Under microgravity conditions, buoyancy-driven stratification is absent and the configuration of the two immiscible liquids is instead governed primarily by interfacial tension and wetting interactions with the container walls. As described in 1.1, 2.3, the polystyrene cuvette walls are preferentially wetted by the oil phase, which results in the oil forming a continuous layer along the boundaries while the water phase occupies the interior of the cuvette.

Consequently, the water phase forms a compact interior domain, surrounded by a continuous oil matrix. The oil–water interface exhibits pronounced curvature and remains smooth and stable prior to activation of the ultrasonic horn, consistent with the capillary-dominated regime (Ca ≪ 1) discussed earlier.

**Fig. 5:**
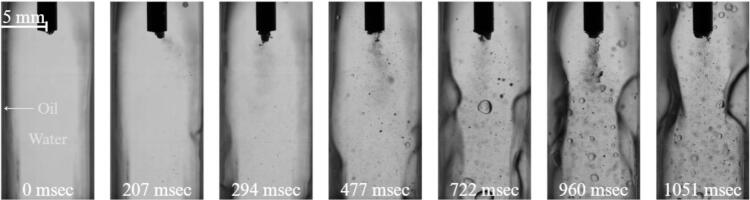
Cavitation and emulsification for 25% oil and 75% water case at 0 g conditions (See also video in Supplementary 2).

After activation of the ultrasonic horn, cavitation bubbles rapidly form at the horn tip and develop into a cavitation cluster similar to that observed in the single-phase water case (Fig. 4). The resulting cavitation stream and associated acoustic streaming establish a recirculating flow within the cuvette (Fig. 5). The downward transport of cavitation bubbles along the stream axis induces compensating upward flow along the side walls, generating a circulating motion within the confined geometry.

In this configuration, the induced flow interacts primarily with the oil matrix attached to the cuvette walls. As shown in Fig. 5, the alternating circulation periodically displaces the oil layer along the left and right walls of the cuvette, leading to temporal variations in its thickness. Despite these deformations, the oil–water interface in most cases remains intact throughout the duration of the experiment. Consequently, the cavitation structures and the associated flow field do not directly penetrate the interface between the two liquids, and the necessary conditions for sustained ultrasonic emulsification are therefore not achieved in this configuration.

### 3.3 50% Oil, 50% water

When equal volumes of oil and water are introduced into the cuvette, the configuration of the two phases differs from the previous cases. Under microgravity conditions, the absence of buoyancy prevents gravitational stratification of the liquids. Due to the capillary forces and wetting interactions with the container walls the polystyrene cuvette walls are preferentially wetted by the oil phase, resulting in the formation of an oil layer along the boundaries while the water phase occupies the interior of the cuvette.

At this oil–water ratio, the interface between the two liquids is located much closer to the ultrasonic horn compared with the previous configuration (Fig. 5). As a consequence, the cavitation structures generated at the horn tip interact more directly with the liquid–liquid interface.

**Fig. 6:**
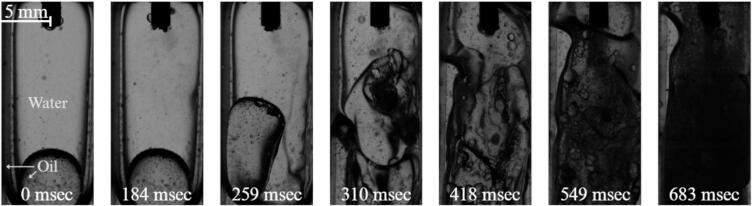
Cavitation and emulsification for 50% oil and 50% water case at 0 g conditions (See also video in Supplementary 3).

Immediately after activation of the ultrasonic horn, cavitation bubbles form at the sonotrode tip and rapidly develop into a dense cavitation cluster. The collapse of these bubbles produces a cavitation stream and associated acoustic streaming similar to that described in the previous sections. However, due to the closer proximity of the interface, the resulting flow field now strongly deforms the oil–water boundary (Fig. 6). The interface begins to oscillate and locally protrude toward the cavitation region. In contrast to the previous configuration, where the interface remained shielded by a continuous oil film along the walls, the cavitation-induced stresses in this case are sufficient to disrupt the interface. Portions of the oil phase are periodically entrained into the water domain, where they are subjected to strong shear and cavitation-induced stresses.

As these oil fragments enter the cavitation zone near the horn tip, they undergo rapid breakup into smaller droplets. Repeated exposure to cavitation bubble collapse and the associated microjets further reduces the droplet size, leading to the formation of a dispersed oil-in-water emulsion. The generated droplets are subsequently transported by the recirculating flow within the cuvette, resulting in a progressively increasing concentration of dispersed oil within the water phase.

One can also note a number of small air bubbles entrained in the oil phase before the beginning of the sonication. These are likely a result of the fact that the cuvette was already exposed to several parabolas before this particular experiment, during which the liquids were lightly mixed, and air bubbles were trapped in the liquid phase. Also possible is that we did not insert the horn into the cuvette carefully enough, trapping come air bubbles beneath it. It is unlikely that these air bubbles would significantly influence the evolution of the experiment once the ultrasonic horn is switched on.

The results demonstrate that when the oil–water interface lies sufficiently close to the cavitation zone, the stresses generated by cavitation collapse and acoustic streaming are capable of continuously disrupting the interface and generating dispersed droplets.

Furthermore, these observations support the conclusion that efficient ultrasonic emulsification requires direct interaction between the cavitation region and the liquid–liquid interface. When this condition is satisfied, the cavitation-driven flow field is able to continuously entrain oil into the water phase and maintain the emulsification process.

### 3.4 75% Oil, 25% water

When the oil fraction is increased to 75%, the configuration of the two phases inside the cuvette changes significantly compared with the previous cases. The water phase now occupies a much smaller volume and forms a compact interior domain surrounded by the oil matrix.

**Fig. 7:**
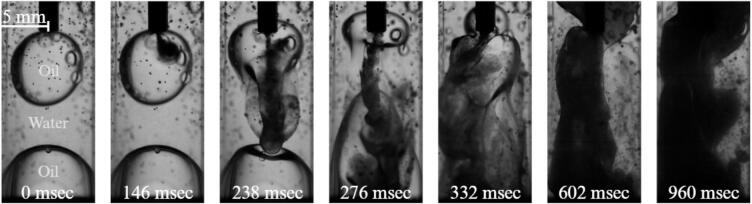
Cavitation and emulsification for 75% oil and 25% water case at 0 g conditions (See also video in Supplementary 4).

In Fig. 7, the initial configuration shows the oil phase separated into two distinct regions prior to activation of the ultrasonic horn. One oil region is in direct contact with the horn tip, while the second is located below, separated by the intervening water phase.

Upon activation of the ultrasonic horn, a cavitation cluster rapidly develops within the oil region adjacent to the sonotrode. As the cavitation cluster reaches a critical density, a directional jet is formed, propagating from the horn tip toward the bottom of the cuvette. During this process, the jet penetrates and disrupts the oil–water interface of the lower oil region. This jet-induced interaction forces the oil from the lower region to detach and migrate upward toward the cavitation zone near the horn tip. As a result, oil is continuously supplied into the region of intense cavitation activity, creating favorable conditions for sustained emulsification.

Once entrained into the cavitation zone, the transported oil undergoes rapid fragmentation due to the combined effects of bubble collapse, microjets, and local shear stresses. Repeated exposure to these mechanisms leads to further droplet breakup and the formation of a fine oil-in-water emulsion. By the end of the process, the mixture appears well dispersed, indicating a high degree of emulsification.

Similarly to the previous case (Fig. 6) one can note a number of small air bubbles entrained in the liquid before the beginning of the sonication. Again these seem not to influence the evolution of the experiment once the ultrasonic horn is switched on.

### 3.5 100% Oil, 0% water

In the final case, the cuvette is filled entirely with silicone oil, and no oil–water interface is present. This configuration serves as a limiting case for evaluating the role of the liquid–liquid interface in the emulsification process.

**Fig. 8:**
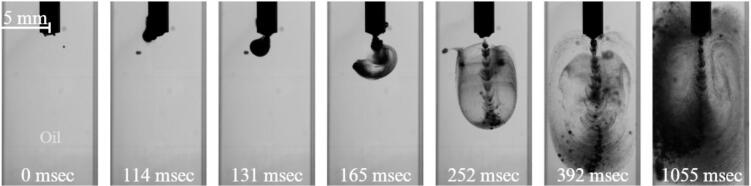
Cavitation in oil at 0 g conditions (See also video in Supplementary 5).

Following activation of the ultrasonic horn, cavitation bubbles form at the sonotrode tip and develop into a cavitation cluster (Fig. 8). Compared with the water-filled case, the cavitation activity is noticeably reduced, which can be attributed to (i) the higher viscosity of the oil phase, which dampens the bubble dynamics, (ii) very low vapor pressure of oil (less than p_v, oil_ < 10 Pa compared to water p_v, water_ = 2340 Pa, both at 20 °C), causing a “delay” in the bubble growth and finally (iii) the higher gas content of oil, which causes more pronounced acoustic attenuation. The combined effects suppresses bubble growth and collapse intensity, resulting in a less pronounced and weaker acoustic streaming.

Despite this, a recirculating flow is still established within the cuvette. As shown in Fig. 8, cavitation bubbles are transported away from the horn tip and circulate within the oil phase, generating localized flow structures similar in topology to those observed in previous cases, albeit with lower intensity.

However, in the absence of a second immiscible phase, no emulsification can occur. While cavitation-induced stresses are present, they do not lead to droplet formation, as there is no liquid–liquid interface to disrupt. The system therefore remains a single-phase flow throughout the experiment.

When examining cavitation in the pure oil case, one can clearly observe the process of degassing and accumulation of undissolved gaseous bubbles in the vicinity of the horn tip. As discussed in 1.1, 2.3, the absence of buoyancy in microgravity fundamentally alters bubble transport. From a nondimensional perspective, as the Bond number approaches zero, gravitational effects vanish and buoyancy-driven rise is suppressed. Under such conditions, bubble motion is no longer governed by density differences, but instead by acoustic radiation forces, diffusion, and weak convective flows.

As a result, undissolved gas accumulates near the ultrasonic horn tip, increasing the local gas nuclei population. This accumulation enhances acoustic attenuation and progressively modifies the cavitation field. In particular, the presence of undissolved bubbles leads to the reorganization of cavitation structures and a reduction in effective acoustic pressure due to shielding effects, as demonstrated recently by Toyran et al. [21]. The observations presented here therefore suggest that gas content and its spatial redistribution could play a critical role in cavitation behavior under microgravity conditions. Consequently, controlled degassing emerges as an important parameter for future investigations, particularly in the context of exploitation of cavitation in reduced-gravity environments.

### 3.6 Emulsification in microgravity and normal gravity conditions

**Fig. 9:**
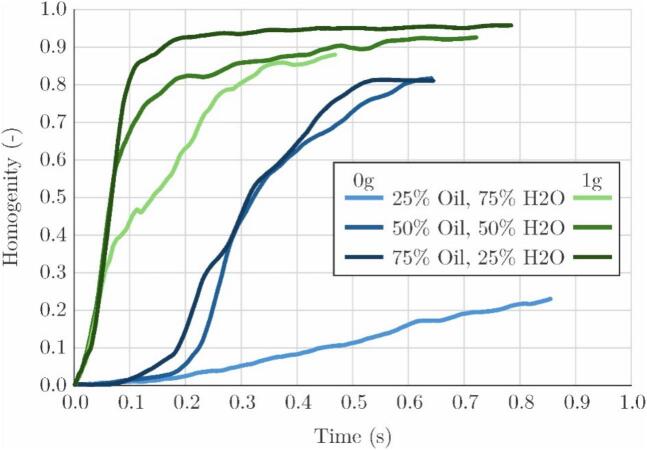
Homogeneity of the sample as a function of sonication time for microgravity and normal gravity conditions and for different oil water ratios.

We compare the experiments performed microgravity (0 g) and normal gravity (1 g) conditions for different oil–water ratios (Fig. 9). The nondimensional parameter of “homogeneity” is determined first by calculating the standard deviation of each sample image in the series. The obtained values were then normalized against the standard deviation of the first image in the series (before the activation of the ultrasonic horn) and finally the, now normalized, value was subtracted from unity. Meaning that in the beginning of the experiment, when clear interfaces between oil and water exist, the image standard deviation is high – homogeneity is low. Later on, when the emulsion is formed the sample is finely mixed, no clear interface exists, standard deviation is low and homogeneity is high.

Fig. 9 shows the results. The vertical axis represents the homogeneity index, which ranges from zero to unity, while the horizontal axis corresponds to time expressed in seconds. Prior to the activation of the ultrasonic horn, all samples exhibit a homogeneity value of zero. At this stage, the silicone oil and distilled water are fully separated into distinct phases. Once the ultrasonic horn is activated and cavitation begins to disrupt the liquid–liquid interface, the two phases start to intermix, and the homogeneity index rises. A higher final value indicates a finer and more uniformly dispersed emulsion, while a steeper initial slope reflects a faster emulsification rate. The homogeneity index therefore serves as a measure of both the speed and the quality of the emulsification process.

Under normal gravity conditions, all three compositions reach high homogeneity values rapidly. The 75% oil mixture achieves the steepest initial rise and plateaus near 0.95, while the 50% and 25% oil mixtures follow closely, reaching final values of approximately 0.92 and 0.87 respectively. The consistently steep gradients across all three 1 g cases indicate that the emulsification process is both fast and efficient under terrestrial conditions regardless of the oil–water ratio tested.

Microgravity conditions, however, lead to a distinctly different outcome. The 75% and 50% oil mixtures still reach appreciable homogeneity values of approximately 0.81, but do so more slowly and with a notably shallower initial slope compared to their 1 g counterparts. Also, a clear “incubation period” can be seen which is related to the time that passes between the activation of the ultrasonic horn and first contact of bubbles and the interface. The most striking difference is observed for the 25% oil mixture, which barely emulsifies over the entire observation window, reaching a homogeneity of only approximately 0.23. This result indicates that at low oil fractions in microgravity, the conditions required for sustained emulsification are rarely met within a reasonable period of time.

These observations are consistent with the interface dynamics described in the preceding sections. Under normal gravity, buoyancy maintains a well-defined and stable liquid–liquid interface that can be reliably positioned within the cavitation zone near the sonotrode tip, ensuring continuous and efficient droplet generation. In microgravity, this stratification is absent and the interface configuration is instead governed by capillarity and wetting. The resulting interface geometry is strongly dependent on the oil–water ratio: at higher oil fractions, the oil coats the cuvette walls and the water occupies a compact interior domain that remains in proximity to the sonotrode tip, preserving the conditions necessary for emulsification. At low oil fractions, however, the oil phase forms only a thin boundary film and the probability of sustained contact between the cavitation zone and the oil–water interface is substantially reduced, leading to the poor emulsification efficiency observed for the 25% oil case in microgravity.

## 4 Conclusions

This study provides a systematic investigation of ultrasonic emulsification under microgravity conditions, with particular emphasis on the role of the oil–water interface and its interaction with cavitation structures. The results demonstrate that the absence of buoyancy fundamentally alters both the spatial organization of immiscible phases and the mechanisms governing emulsification.

In contrast to normal gravity conditions, where buoyancy stabilizes a well-defined interface that can be effectively positioned within the cavitation zone, microgravity leads to capillarity-driven configurations in which the interface becomes less predictable and more difficult to control. As a consequence, sustained interaction between the cavitation region and the liquid–liquid interface is not always achieved, which directly reduces emulsification efficiency. This is consistently reflected in the temporal evolution of the homogeneity index, where microgravity cases exhibit slower emulsification rates and lower final homogeneity values.

The experimental results further confirm that a direct and continuous link between the cavitation zone and the interface is the key condition for effective emulsification, regardless of the gravity regime. When this condition is satisfied, cavitation-induced stresses, microjets, and acoustic streaming enable efficient droplet breakup and dispersion. However, in microgravity, achieving this link becomes a challenge due to the absence of buoyancy-driven positioning mechanisms.

In addition, the pure oil case highlights an important secondary effect: the accumulation of undissolved gases near the ultrasonic horn. Under microgravity conditions, where buoyancy-driven bubble removal is suppressed, gas transport is governed primarily by acoustic and diffusive mechanisms, leading to local undissolved gas enrichment. This accumulation modifies the cavitation field through shielding effects, reduces effective acoustic pressure, and alters cavitation structure dynamics, as also observed in our previous study [21]. These findings indicate that gas content and its spatial distribution represent critical, yet often overlooked, parameters in microgravity cavitation systems.

Future work should focus on active control strategies, particularly controlled degassing and interface positioning techniques. Such approaches are expected to play a crucial role in enabling reliable and efficient emulsification processes in space environments, with direct relevance to fluid management, material processing, and life-support systems in long-duration missions.

## CRediT authorship contribution statement

**Jakob Mali:** Writing – original draft, Visualization, Methodology, Investigation, Funding acquisition, Conceptualization. **Žan Boček:** Writing – original draft, Visualization, Methodology, Investigation, Formal analysis, Conceptualization. **Jernej Ortar:** Writing – original draft, Methodology, Investigation, Conceptualization. **Sara Poropatič:** Writing – original draft, Visualization, Conceptualization. **Leon Čepin:** Methodology, Conceptualization. **Nejc Bertoncelj:** Software, Methodology, Conceptualization. **Matevž Dular:** Writing – original draft, Visualization, Validation, Supervision, Methodology, Investigation, Funding acquisition, Formal analysis, Conceptualization.

## Declaration of competing interest

The authors declare that they have no known competing financial interests or personal relationships that could have appeared to influence the work reported in this paper.

## Appendix A Supplementary data

Supplementary video 1 Cavitation in water at 0 g conditions (only every 10^th^ image showed).

Supplementary video 2 Cavitation and emulsification for 25% oil and 75% water case at 0 g conditions (only every 10^th^ image showed).

Supplementary video 3 Cavitation and emulsification for 50% oil and 50% water case at 0 g conditions (only every 10^th^ image showed).

Supplementary video 4 Cavitation and emulsification for 75% oil and 25% water case at 0 g conditions (only every 10^th^ image showed).

Supplementary video 5 Cavitation in oil at 0 g conditions (only every 10^th^ image showed).
